# Mutant p53 confers chemoresistance in non-small cell lung cancer by upregulating Nrf2

**DOI:** 10.18632/oncotarget.6150

**Published:** 2015-10-19

**Authors:** Min-Che Tung, Po-Lin Lin, Yao-Chen Wang, Tsung-Ying He, Ming-Ching Lee, Sauh-Der Yeh, Chih-Yi Chen, Huei Lee

**Affiliations:** ^1^ Graduate Institute of Clinical Medicine, Taipei Medical University, Taipei, Taiwan; ^2^ Graduate Institute of Cancer Biology and Drug Discovery, Taipei Medical University, Taipei, Taiwan; ^3^ Department of Surgery, Tungs' Taichung MetroHarbor Hospital, Taichung, Taiwan; ^4^ Institute of Medicine, Department of Surgery, Chung Shan Medical University, Taichung, Taiwan; ^5^ Division of Thoracic Surgery, Department of Surgery, Chung Shan Medical University, Taichung, Taiwan; ^6^ Division of Chest Medicine, Chung Shan Medical University Hospital, Taichung, Taiwan; ^7^ Department of Thoracic Surgery, Taichung Veteran General Hospital, Taichung, Taiwan

**Keywords:** Nrf2, p53, cisplatin sensitivity, lung cancer

## Abstract

Nrf2 is a key transcription factor for genes coding for antioxidants, detoxification enzymes, and multiple drug resistance and it also confers resistance to anticancer drugs. Here, we hypothesized that mutant p53 could upregulate Nrf2 expression at the transcriptional level, thereby conferring cisplatin resistance in non-small cell lung cancer (NSCLC). Luciferase reporter assays and real-time PCR analysis indicated that the Nrf2 promoter activity and its mRNA levels were markedly suppressed by wild-type p53, but not by mutant p53. Chromatin immunoprecipitation (ChIP) further confirmed that wild-type p53 binds at the p53 putative binding site to block Sp1 binding to the Nrf2 promoter and consequently to suppress the Nrf2 promoter activity. The MTT assay indicated that an increase in Nrf2 expression by mutant p53 is responsible for cisplatin resistance. Among the Nrf2 downstream genes, Bcl-2 and Bcl-xL contribute more strongly to Nrf2-mediated cisplatin resistance when compared with heme oxygenase 1 (HO-1). Cox regression analysis showed that patients with high-Nrf2, high-Bcl-2, high-Bcl-xL mRNA tumors were more commonly occurred unfavorable response to cisplatin-based chemotherapy than their counterparts. The prognostic significance of Nrf2 mRNA levels on OS and RFS was also observed in patients who have received cisplatin-based chemotherapy, particularly in p53-mutant patients. Collectively, mutant p53 may confer cisplatin resistance via upregulation of Nrf2 expression, and Nrf2 mRNA level may predict chemotherapeutic response and outcomes in NSCLC.

## INTRODUCTION

Lung cancer is the leading cause of cancer death around the world. Late diagnosis and low response to therapeutic drugs are viewed as the causes of poor patient outcomes [1]. The five-year survival rate has remained at about 15% for the past three decades despite the development and use of several targeting drugs for lung cancer therapy [2]. Therefore, mechanistic studies to uncover the possible pathway(s) involved in drug resistance are essential for improving the outcomes and life quality in patients with this disease.

Cisplatin-based chemotherapy is still considered the first-line therapeutic strategy for lung cancer [3, 4]. Cisplatin induces cancer cell death *via* induction of double strand DNA breaks caused by the generation of reactive oxygen species (ROS) [5, 6]. Gene expression of antioxidants that eliminate ROS is promoted by an NF-E2-related factor 2 (Nrf2), which binds to antioxidant response elements (ARE) of the promoters of these genes [7–11]. Activation of Nrf2/ARE signaling has been demonstrated through mutations of Kelch-like ECH-associated protein 1 (Keap1) or Nrf2 to protect Nrf2 from degradation by Keap1 [12–17]. The Keap1/Nrf2 mutations in NSCLC patients were ranged from 3.2% to 60% and this variation may be due to the number of study subjects and histologic subtypes. In fact, the Keap1/Nrf2 mutation was frequently reported to be uncommon in NSCLC patients including Taiwanese (< 3%); (Supplementary Table 1). High Nrf2 expression has been shown to promote resistance to different anticancer drugs in human cancers [18–22]. However, the underlying mechanism of an increase in Nrf2 expression is not fully understood although some mechanisms have been reported [23, 24]. For example, high Nrf2 expression driven by NF-κB signaling confers chemoresistance in human myeloid leukemia [23]. Mutant k-ras confers chemoresistance by upregulating Nrf2 transcription through a TPA response element [24].

The cross-talk between Nrf2 and p53 plays a crucial role in cellular homeostasis. Positive or negative co-regulation at the post-translational level has been reported between Nrf2 and p53 [25]. For example, binding of the p53 downstream p21 to Nrf2 inhibits Nrf2 degradation [26], whereas binding of the Nrf2 downstream NAD(P)H dehydrogenase quinone 1 (NQO1) to p53 prevents p53 degradation [27]. However, Nrf2 promotes MDM2 expression for degradation of p53 protein [28]. P53 inhibits Nrf2 downstream expression of genes, such as NQO1 and heme oxygenase 1 (HO-1) by directly interacting with ARE-containing promoters [29]. Therefore, we expected that the reciprocal regulation between Nrf2 and p53 may modulate cancer cell apoptosis induced by chemotherapeutic agents.

Our preliminary data from lung cancer patients showed that Nrf2 mRNA expression levels were higher in p53-mutant tumors than in p53-wild-type tumors. A software analysis revealed that putative binding sites for Sp1 and p53 could exist on the Nrf2 promoter (−1036/+1). We therefore hypothesized that mutant p53 might directly upregulate Nrf2 transcription to confer resistance of chemotherapeutic agents and consequently result in poor outcome in NSCLC patients.

## RESULTS

### Nrf2 expression is suppressed by wild-type p53, but not by mutant p53 at the transcription level

**Figure 1:**
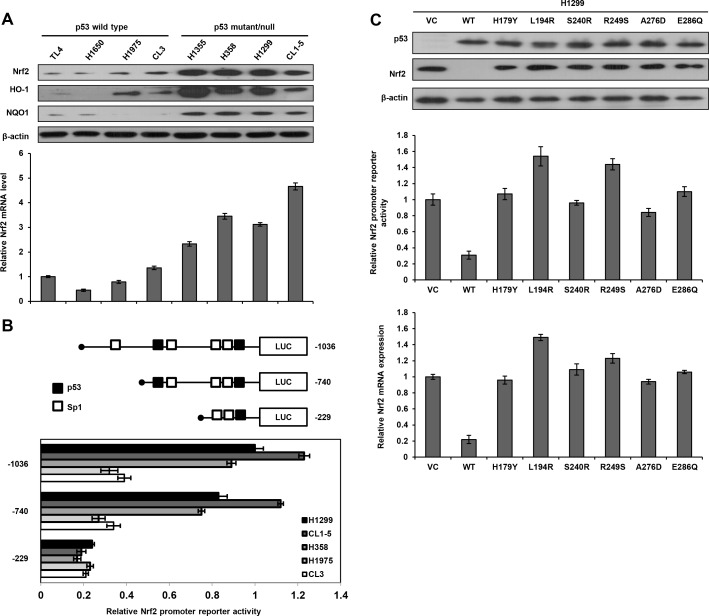
Nrf2 transcription is suppressed by wild-type p53 **A.** Western blotting analysis for Nrf2, HO-1, and NQO1 expression in various lung cancer cell lines, β-actin was used as a loading control. Real-time PCR analysis was performed to determine Nrf2 mRNA expression; GAPDH was served as an internal control. Nrf2 mRNA expression in TL4 cells (mRNA level = 1) was used as a reference to evaluate Nrf2 mRNA levels in other lung cancer cells. **B.** Diagram summarizing the positions of the p53 and Sp1 putative binding sites on Nrf2 promoter constructs (−1036∼+1) predicted by a software analysis (http://www.atcc.org). An luciferase reporter assay was performed to evaluate the reporter activity of these three promoter fragments, including −1036/+1, −740/+1, and −229/+1. H1299, CL1-5, H358, H1975, and CL3 cells were separately transfected with these three promoters (5μg) for the luciferase reporter assay; β-gal was served as an internal control. The reporter activity of the Nrf2 (−1036/+1) promoter in H1299 cells served as a reference (activity = 1). **C.** Number of p53 wild-type/mutants plasmid and Nrf2 promoters plasmid (−740/+1) were co-transfected into H1299 cells, the cells lysates were separated by SDS-PAGE for the evaluation p53 expression by a specific antibody using western blotting. Luciferase reporter assay was performed to evaluate the reporter activity of Nrf2 promoter. Real-time PCR analysis was performed to determine Nrf2 mRNA expression.

Eight lung cancer cell lines were enrolled to test the hypothesis that Nrf2 expression is de-regulated at the transcription level by p53 status. The Nrf2/ARE downstream genes HO-1 and NQO1 expression were relatively lower in p53-wild-type cells than in p53-mutant cells (Figure 1A upper panel). Concomitantly, Nrf2 protein and its mRNA expression levels were significantly lower in p53-wild-type cells than in p53-mutant cells (Figure 1A). These results suggest that Nrf2 expression might be suppressed by wild-type p53, but not by mutant p53 at transcriptional level.

A software analyses predicted two p53 and four Sp1 putative binding sites located on the −1036/−1 Nrf2 promoter (http://www.genome.jp/tools/motif/). Three Nrf2 promoters (−1036/+1, −740/+1, and −229/+1) were constructed by PCR and deletion mutations for luciferase reporter assay (Figure 1B upper panel). These three Nrf2 promoters were respectively transfected into H1299, H1975, and CL3 cells. The reporter activity was significantly higher for the −1036/+1 Nrf2 promoter than for the other two Nrf2 promoters (−740/+1 and −229/+1) in these cells. These results revealed the possibility that p53 and Sp1 putative binding sites located near the transcriptional start site might play an important role in Nrf2 transcription in these three cell types.

The possibility that wild-type p53 could suppress Nrf2 promoter activity was explored using p53-null H1299 cells by transfection with wild-type p53 or different mutant p53 expression vectors including H179Y, L194R, S240R, R249S, A276D, and E286Q. Western blotting showed that p53 expression was detected in H1299 cells transfected with wild-type or mutant p53 expression vectors, but was not observed in H1299 cells with an empty vector transfection (VC) (Figure 1C upper panel). Nrf2 expression was almost completely suppressed by wild-type p53 expression vector, but was unchanged by mutant p53 expression vector transfections in H1299 cells. The luciferase reporter assay indicated a marked decrease in Nrf2 promoter activity following wild-type p53 expression vector transfection, but showed unchanged or relatively increased Nrf2 promoter activity following transfection with mutant p53 expression vectors when compared with the activity in VC cells (Figure 1C lower panel). These results suggest that Nrf2 expression in lung cancer cells is suppressed by wild-type p53, but not by mutant p53 at the transcriptional level.

### Nrf2 transcription is down-regulated by wild-type p53, but not by mutant p53 *via* decreased Sp1 binding to the Nrf2 promoter

**Figure 2:**
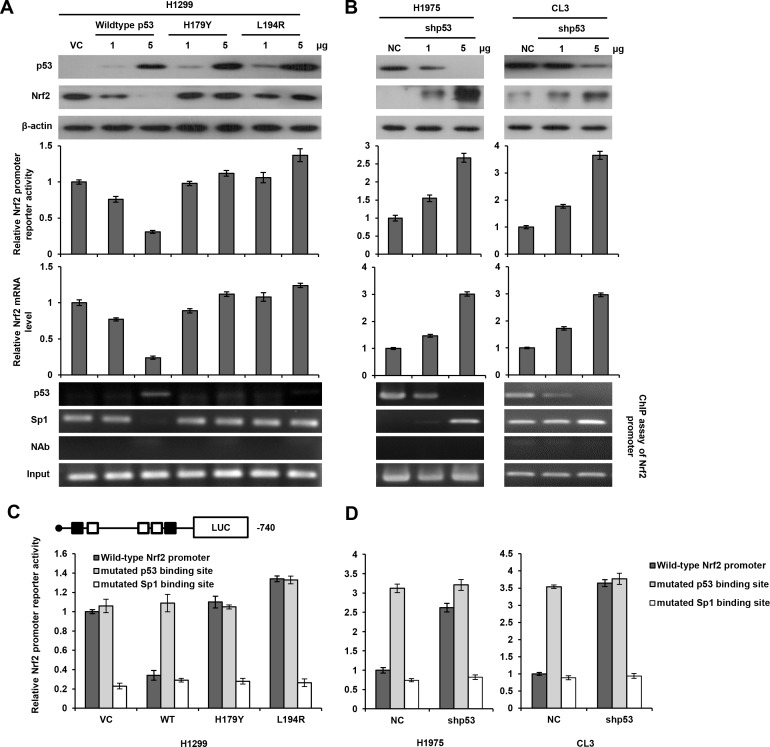
Nrf2 transcription is down-regulated by wild-type p53 via decreased Sp1 binding to the Nrf2 promoter **A.** The Nrf2 (−740/+1) promoter activity in H1299 cells, which were transfected with p53 wild-type/mutants plasmid. The cells lysates were separated by SDS-PAGE for the evaluation p53 and Nrf2 expression by specific antibodies using western blotting. An luciferase reporter assay was performed to evaluate the reporter activity of Nrf2 promoter. A ChIP assay was performed to evaluate the binding ability of p53 and Sp1 to the putative binding site of the Nrf2 promoter region. The products were amplified by PCR and the as gel electrophoresis results are presented. **B.** The reporter activity of the Nrf2 (−740/+1) promoter in H1975 and CL3 cells, which were transfected with shp53 plasmid. The cells lysates were separated by SDS-PAGE for the evaluation p53 and Nrf2 expression by specific antibodies using western blotting. Luciferase reporter assay was performed to evaluate the reporter activity of Nrf2 promoter. A ChIP assay was performed to evaluate the binding ability of p53 and Sp1 to the putative binding site of the Nrf2 promoter region. The products were amplified by PCR and the gel electrophoresis results are presented. **C.** The reporter activity of the wild-type-, p53 binding site mutated-, and Sp1 binding site mutated-Nrf2 (−740/+1) promoter in H1299, which were transfected with p53 wild-type/mutants plasmid. An luciferase reporter assay was performed to evaluate the reporter activity of Nrf2 promoter. **D.** The reporter activity of the wild-type-, p53 binding site mutated-, and Sp1 binding site mutated-Nrf2 (−740/+1) promoter in H1975 and CL3 cells, which were transfected with shp53 plasmid. An luciferase reporter assay was performed to evaluate the reporter activity of Nrf2 promoter.

We next examined the possibility that wild-type p53 could interact with Sp1 to suppress Nrf2 transcription. The p53-null H1299 cells were transfected with two doses of wild-type p53 or mutant p53 expression vectors (H179Y and L194R). The expression of p53 protein in H1299 cells with wild-type or mutant p53 expression vector transfection was revealed by western blotting, but Nrf2 expression was almost completely suppressed by a high dose of wild-type p53 transfection, but was nearly unchanged by mutant p53 expression vector transfections in H1299 cells (Figure 2A upper panel). The luciferase reporter assay and real-time PCR analysis revealed a dose-dependent decrease in Nrf2 promoter activity and its mRNA levels in response to wild-type p53 transfection in H1299 cells. However, Nrf2 promoter activity and its mRNA level were nearly unchanged in H1299 cells transfected with H179Y or L194R expression vector, whereas Nrf2 promoter activity was elevated by high transfection doses of L194R expression vector (Figure 2A middle panel). ChIP assay showed that wild-type p53 was indeed bound, but the binding of mutant p53 was not revealed on the p53 binding site of Nrf2 promoter. The binding of Sp1 to the Nrf2 promoter in H1299 cells with wild-type p53 transfection almost completely eliminated when compared with VC and H1299 cells transfected with mutant p53 expression vectors (Figure 2A lower panel). On the other hand, Nrf2 protein, Nrf2 promoter activity, and its mRNA expression were elevated by p53 knockdown in p53-wild-type H1975 and CL3 cells. The binding of p53 and Sp1 to the Nrf2 promoter was decreased and increased by p53 silencing in both cell types (Figure 2B). A ChIP analysis further indicated that the binding of p53 to the Nrf2 promoter was decreased by p53 silencing, but the binding of Sp1 to the Nrf2 promoter was increased in p53-knockdown H1975 and CL3 cells in a dose-dependent manner (Figure 2B lower panel). These results suggest that p53 may interfere Sp1 binding to the Nrf2 promoter to reduce its promoter activity.

We then constructed Nrf2 promoters mutated at the p53 or Sp1 binding site by site-directed mutagenesis to verify whether wild-type p53 could interact with Sp1 to suppress Sp1 binding to the Nrf2 promoter. The Nrf2 promoter activity was unchanged by transfection with an Nrf2 promoter mutated at the p53 binding site in H1299 VC cells and H1299 cells with mutant p53 expression vector transfections, but the promoter activity was significantly elevated in H1299 cells transfected with the Nrf2 wild-type promoter. However, the Nrf2 promoter activity was elevated by Nrf2 wild-type promoter transfection in mutant p53-tranfected H1299 cells, but this promoter activity was completely eliminated by transfection with an Nrf2 promoter mutated at the Sp1 binding site in H1299 cells subjected to different treatments (Figure 2C). On the other hand, the Nrf2 promoter activity was markedly increased by transfection with an Nrf2 promoter mutated at the p53 binding site, but the increase in the Nrf2 promoter activity in p53-knockdown H1975 and CL3 cells was nearly completely reversed by transfection with an Nrf2 promoter mutated in the Sp1 binding site (Figure 2D). These results clearly indicate that Nrf2 transcription in lung cancer cells is predominately down-regulated by wild-type p53 *via* decreased Sp1 binding to the Nrf2 promoter, but is not affected by mutant p53.

### Nrf2-mediated Bcl-2, Bcl-xL, and HO-1 expression is dependent on p53 status and may confer cisplatin resistance

**Figure 3:**
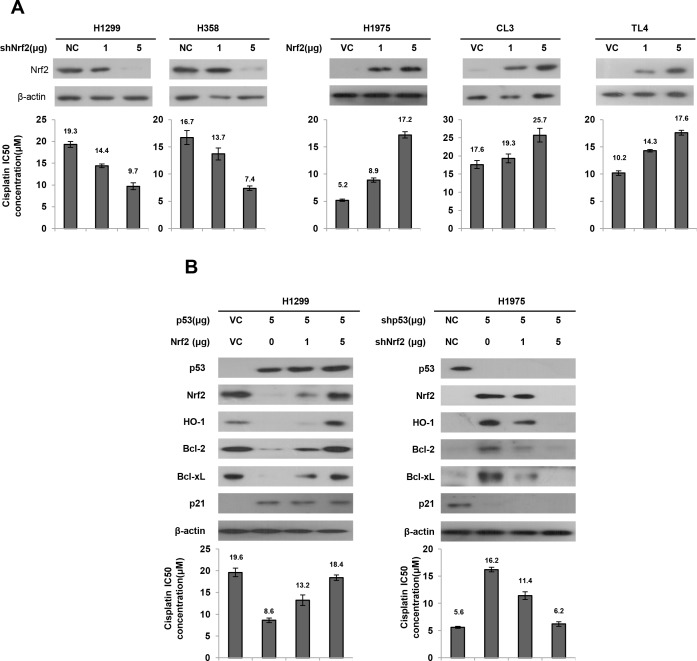
Nrf2 expression is responsible for cisplatin sensitivity in lung cancer cells **A.** shNrf2 plasmids were transfected into high Nrf2-expressing (H1299 and H358) cell lines compared with both cell types transfected with a non-specific shRNA (NC), Nrf2 expression plasmids were transfected into low Nrf2-expressing (H1975 and CL3) cell lines compared with both cell types transfected with an empty vector (VC). After 24h, the indicated cells were incubated with or without cisplatin (0, 2, 4, 8, 16, 32 μM) for 48 h for MTT assay. The cell lysates were separated by SDS-PAGE for the evaluation Nrf2 expression by specific antibodies using western blotting. The MTT assay was used to determine the 50% inhibition concentration (IC50) of cisplatin. **B.** H1299 cells were transfected with p53 and/or Nrf2 plasmid. H1975 cells were transfected with shp53 and/or shNrf2 plasmid. After 24h, the indicated cells were incubated with or without cisplatin for 48 h for MTT assay. The cell lysates were separated by SDS-PAGE for the evaluation p53, Nrf2, HO-1, Bcl-2 and Bcl-xL expression by specific antibodies using western blotting. The MTT assay was used to determine the IC50 of cisplatin.

We investigated whether Nrf2 expression could determine cisplatin sensitivity in lung cancer cells that had different p53 status. Two types of p53-null cells (H1299 and H358) and three types of p53-wild-type cells (H1975, CL3, and TL4) were collected to evaluate the inhibitory concentration yielding 50% cell viability (IC50) for cisplatin using the MTT assay. Western blotting showed that Nrf2 expression was decreased by Nrf2 knockdown in H1299 and H358 cells, but was dose-dependently increased by Nrf2 overexpression in H1975, CL3, and TL4 cells (Figure 3A upper panel). Intriguingly, the IC50 value for cisplatin was concomitantly decreased by Nrf2-knockdown in H1299 and H358 cells, but was increased in Nrf2-overexpressing H1975, CL3, and TL4 cells (Figure 3A lower panel). However, the IC50 value for cisplatin was nearly unchanged by different mutant p53 expression vector transfections in H1299 cells when compared with VC cells; however, the IC50 value for cisplatin was almost completely rescued by Nrf2 knockdown in H1299 cells transfected with different mutant p53 expression vectors (Supplementary Figure 1). These results clearly indicated that Nrf2 expression is dependent on p53 status and may confer cisplatin resistance in lung cancer cells.

Nrf2 upregulates Bcl-2 and Bcl-xL transcription and in turn confers drug resistance [22, 30]. HO-1 is a downstream gene of the Nrf2/ARE signaling that promotes tumor drug resistance [31–33]. The possibility that wild-type p53-mediated Nrf2 reduction could promote cisplatin sensitivity was explored by transfecting wild-type p53 into H1299 cells. Western blotting indicated that Nrf2 expression was nearly completely eliminated by wild-type p53 transfection in H1299 cells when compared with VC cells (Figure 3B upper panel). As expected, the decrease in Nrf2 expression in wild-type p53-transfected H1299 cells was gradually increased by ectopic expression of Nrf2. As expected, p53-downstream p21 expression was increased by wild-type p53 overexpression in H1299 cells, but was decreased by wild-type p53 knockdown in H1975 cells. The expression of Bcl-2, Bcl-xL, and HO-1 was concomitantly elevated by ectopic expression of Nrf2 in wild-type p53-transfected H1299 cells. The IC50 value for cisplatin in H1299 cells was markedly decreased by wild-type p53 transfection, but the IC50 value was gradually increased by co-transfection with Nrf2 expression vector in H1299 cells (19.6 μM *vs*. 8.6 μM *vs*. 13.2 μM *vs*. 18.4 μM; Figure 3B left lower panel). The increase in Nrf2, Bcl-2, Bcl-xL, and HO-1 expressions were observed in p53-knockdown H1975 cells, but the increases of these three molecules were reversed by Nrf2 silencing in p53-knockdown H1975 cells (Figure 3B, right upper panel). The IC50 value of p53-knockdown H1975 cells was increased to 16.2 μM when compared with H1975 cells with non-specific shRNA transfection (NC) (5.6 μM). However, the increase in the IC50 value in p53-knockdown H1975 cells was suppressed by Nrf-2 silencing in a dose-dependent manner (Figure 3B, right lower panel). Annexin-V/PI assay further indicated that the percentages of cell apoptosis in both cell types were elevated and reduced by p53 manipulation (Supplementary Figure 2). These results suggest that Nrf2-mediated Bcl-2, Bcl-xL and HO-1 is dependent on p53 status and may confer cisplatin resistance *via* apoptotic machinery.

### Bcl-2 and Bcl-xL are more involved than HO-1 in Nrf2-mediated cisplatin resistance

**Figure 4:**
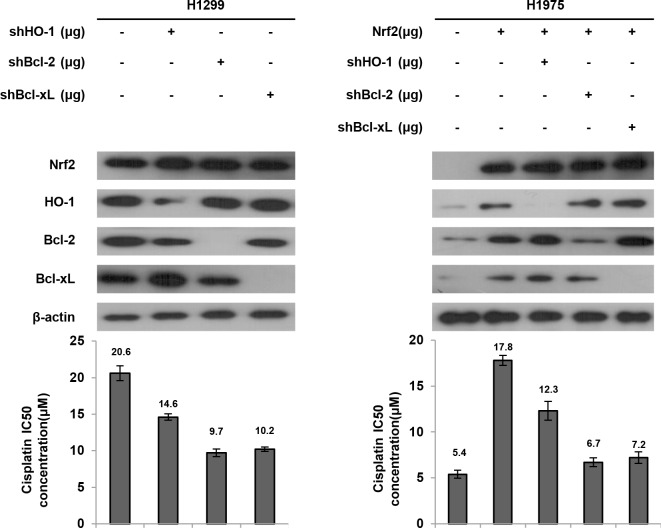
Bcl-2 and Bcl-xL are more involved than HO-1 on Nrf2-mediated cisplatin resistance H1299 cells were transfected with shHO-1, shBcl-2 or shBcl-xL plasmid. H1975 cells were transfected with Nrf2, shHO-1, shBcl-2 or shBcl-xL plasmid. After 24h, the indicated cells were incubated with or without cisplatin for 48 h for MTT assay. The cell lysates were separated by SDS-PAGE for the evaluation Nrf2, HO-1, Bcl-2 and Bcl-xL expression by specific antibodies using western blotting. The MTT assay was used to determine the IC50 of cisplatin.

We next examined which genes regulated by Nrf2 were more involved in cisplatin resistance. High Nrf2 expressing H1299 cells were selected for transfection with shHO-1, shBcl-2, or shBcl-xL, and low Nrf2 expressing H1975 cells were transfected with the Nrf2 expression vector and then co-transfected with shHO-1, shBcl-2, or shBcl-xL. The MTT assay results showed that the IC50 value of H1299 and Nrf2-overexpressing H1975 cells was markedly decreased by shHO-1, shBcl-2, or shBcl-xL, when compared with H1299 NC cells and Nrf2-overexpressing H1975 cells (Figure 4). Interestingly, the IC50 value was lower for both cell types transfected with shBcl-2 or shBcl-xL than for both cell types transfected with shHO-1 (Figure 4). These results clearly indicated that Bcl-2 and Bcl-xL are more involved than HO-1 in Nrf2-mediated cisplatin resistance.

### Nrf2 mRNA levels are associated with p53 status and related to tumor responses to cisplatin-based chemotherapy in NSCLC patients

We examined whether Nrf2 mRNA expression levels could be associated with p53 status in NSCLC patients. In total, 114 tumors from surgically resected NSCLC patients, who were determined not to have keap1 and Nrf2 mutation (*n* = 109, Supplementary Table 1), were evaluated for Nrf2 mRNA expression levels using real-time PCR. The median value of Nrf2 mRNA expression levels in lung tumors was used as a cutoff point to divide patients into high-Nrf2 and low-Nrf2 subgroups and the categories were further confirmed by a box plot analysis (Supplementary Figure 3). The p53 mutation data were obtained from our previous reports [34, 35]. Nrf2 mRNA expression was not associated with clinico-pathological parameters, including age, gender, smoking status, tumor histology, and stage. Interestingly, high-Nrf2 mRNA tumors were more commonly observed in p53-mutant patients than in p53-wild-type patients (70.8% *vs*. 43.5%, *p* = 0.018; Table 1). High Bcl-2 and high Bcl-xL mRNA expression were more prevalently occurred in high-Nrf2 mRNA tumors than in low-Nrf2 mRNA tumors (59.3% *vs*. 40%, *p* = 0.044 for Bcl-2; 61.1% *vs*. 38.2%, *p* = 0.017; Table 1).

**Table 1:** Relationships between Nrf2 mRNA expression and clinical-pathological parameters NSCLC patients

Characteristic	Case no.	Nrf2 mRNA		*P*
		Low (%)	High (%)	
	109	55(50.5)	54(49.5)	
Age				
≦66	56	28(50.0)	28(50.0)	0.708
＞66	53	27(50.9)	26(49.1)	
Gender				
Female	37	21(56.8)	16(36.8)	0.346
Male	72	34(47.2)	38(52.8)	
Smoking status				
Nonsmoking	61	35(57.4)	26(49.0)	0.103
Smoking	48	20(41.7)	28(58.3)	
Tumor type				
AD	67	37(55.2)	30(44.8)	0.209
SQ	42	18(42.9)	24(57.1)	
Stage				
I	38	20(52.6)	18(47.4)	0.423
II	19	7(36.8)	12(63.2)	
III	52	28(53.8)	24(46.2)	
p53 mutation				
No	85	48(56.5)	37(43.5)	0.018
Yes	24	7(29.2)	17(70.8)	
Bcl-2				
Low	55	33(60.0)	22(40.0)	0.044
High	54	22(40.7)	32(59.3)	
Bcl-xL				
Low	55	34(61.8)	21(38.2)	0.017
High	54	21(38.9)	33(61.1)	

We next examined the possibility that Nrf2 mRNA expression levels could be associated with the tumor response to cisplatin-based chemotherapy. In total, 60 of the 109 patients were available for this retrospective study, and data indicated that an unfavorable response to cisplatin-based chemotherapy was more likely in patients with high-Nrf2 mRNA tumors than with low-Nrf2 mRNA tumors (71.9% *vs*. 21.4%, *p* = 0.001; Table 2). Similar findings in an unfavorable response to cisplatin-based chemotherapy were more commonly observed in high-Bcl-2 or high-Bcl-xL mRNA tumors than their counterparts (62.5% *vs*. 32.1% for Bcl-2; 66.7% *vs*. 33.3%, *p* = 0.01; Table 2). We further showed that an unfavorable response to cisplatin-based chemotherapy was more common in p53-mutant patients who harbored high-Nrf2 mRNA tumors than low-Nrf2 tumors (70% *vs*. 15.8%, *p* = 0.004; Table 2). These results suggested that high-Nrf2 mRNA patients whose tumors harbored p53 mutations may more frequently show an unfavorable response to cisplatin-based chemotherapy when compared with patients whose tumors harbored wild-type p53. Bcl-2 and Bcl-xL expression may be partially contributive to Nrf2-mediated unfavorable response to cisplatin-based chemotherapy in NSCLC patients.

**Table 2:** Association between Nrf2, Bcl-2 and Bcl-xL mRNA in lung tumors and tumor response to cisplatin-based chemotherapy in NSCLC patients with tumor recurrence and/or metastasis after surgical resection

Characteristic		Tumor Response		*P*
		Unfavorable (%)	Favorable (%)	
		29(48.3)	31(51.7)	
Nrf2				
Low	27	6(22.2)	21(77.8)	0.001
High	33	23(69.7)	10(30.3)	
Bcl-2				
Low	28	9(32.1)	19(67.9)	0.019
High	32	20(62.5)	12(37.5)	
Bcl-xL				
Low	33	11(33.3)	22(66.7)	0.010
High	27	18(66.7)	9(33.3)	
p53/Nrf2				
WT/Low	19	3(15.8)	16(84.2)	0.004
Mutation/High	10	7(70.0)	3(30.0)	

### Nrf2 mRNA expression levels are associated with overall survival (OS) and relapse free survival (RFS) in NSCLC patients

We next examined the possibility that Nrf2 mRNA expression levels could be associated with OS and RFS in NSCLC patients. Cox regression analysis using all studied population (*n* = 109) indicated that high-Nrf2 mRNA patients exhibited worse OS and RFS than low-Nrf2 mRNA patients (hazard ratio, HR, 2.014, 95% CI, 1.03-3.87, *p* = 0.013 for OS; HR, 2.047, 95% CI, 1.17-4.069, *p* = 0.022 for RFS; Table 3). The five-year survival rate and median survival month for OS and RFS were lower and shorter in high-Nrf2 mRNA patients than in low-Nrf2 mRNA patients (OS: 12.3% *vs*. 36.8% for five-year survival rate, 22.3 months *vs*. 45.3 months; RFS: 3.9% *vs*. 14.8% for five-year survival rate, 13.4 months *vs*. 22.3 months).

**Table 3:** Cox regression analysis for Nrf2 mRNA, p53 status, and combining Nrf2 mRNA with p53 status on OS and RFS in NSCLC patients

	OS						RFS					
	Case no.	5- year survival (%)	Median survival (month)	HR	95% CI	P	Case no.	5- year survival (%)	Median survival (month)	HR	95% CI	P
	109						98					
Nrf2 mRNA												
Low	55	36.8	45.3	1			47	14.8	22.3	1		
High	54	12.3	22.3	2.014	1.03-3.87	0.013	51	3.9	13.4	2.047	1.17-4.069	0.022
p53 status												
Wild type	85	22.7	25.4	1			78	10.3	12.6	1		
Mutation	24	30.8	33.9	0.785	0.42-1.31	0.351	20	15.0	21.2	0.842	0.39-1.21	0.521
Nrf2 mRNA (chemo)												
Low	28	37.4	46.0	1			27	11.1	21.0	1		
High	32	5.4	21.0	2.203	1.11-4.36	0.023	29	0.0	16.9	1.992	1.10-3.93	0.047
p53 status (chemo)												
Wild type	42	21.6	20.7	1			40	10.8	17.2	1		
Mutation	18	26.1	35.3	0.644	0.32-1.28	0.208	16	6.3	28.5	0.706	0.36-1.40	0.319
p53/Nrf2												
WT/Low	48	41.1	32.7	1			35	25.7	14.1	1		
Mutation/High	17	32.7	22.4	1.758	0.82-3.79	0.151	12	0.0	12.9	2.269	1.02-5.07	0.046

HR: adjusted by stage.

Chemo: the prognostic value of Nrf2 mRNA, p53 status, and combining Nrf2 mRNA with p53 status in patients who received cisplatin-based chemotherapy.

A prognostic significance of Nrf2 mRNA expression levels on OS and RFS was still observed in 60 patients who have received cisplatin-based chemotherapy (HR, 2.203, 95% CI, 1.11-4.36, *p* = 0.023 for OS; HR, 1.992, 95% CI, 1.10-3.93, *p* = 0.047; Table 3). However, a prognostic significance of p53 status on OS and RFS was not revealed in all studied cases or in the 60 patients who had received cisplatin-based chemotherapy (Table 2). We also found worse RFS in p53-mutant patients who harbored high-Nrf2 tumors rather than low-Nrf2 mRNA tumors (HR, 2.269, 95% CI, 1.02-5.07, *p* = 0.046; Table 3), but a prognostic value for OS was not observed in the high-Nrf2 patients. The chemotherapeutic regimens for these sixty patients are listed in Supplementary Table 2. These patients received cisplatin alone (8.3%) and/or combined with other chemotherapeutic agents including gemcitabine (73.3%), vp16 (8.3%), taxol (8.3%), and mitomycin C (1.7%). These results suggest that high Nrf2 mRNA expression levels may be useful for prediction of poorer OS and RFS in NSCLC patients. A prognostic significance of Nrf2 mRNA levels on OS and RFS was also observed in patients who had received cisplatin-based chemotherapy.

## DISCUSSION

The present study provides evidence that suppression of Nrf2 transcription by wild-type p53, occurring *via* decreased Sp1 binding to the Nrf2 promoter, may confer cisplatin sensitivity, a favorable chemo-response, thereby leading to favorable outcomes in lung cancer patients. Moreover, a decrease in Nrf2 mRNA expression by wild-type p53 corresponded with its protein expression. These findings suggest that Nrf2 expression is predominately regulated by wild-type p53 at the transcription level. Conversely, Nrf2 mRNA and its protein expression levels in H1299 cells were markedly elevated by different mutant p53 expression vector transfections when compared with VC cells (Figure 1C). A previous report has indicated that Nrf2 expression is driven by the NF-κB signaling pathway in acute myeloid leukemia [23]. Mutation of p53 gene prolongs NF-κB activation and promotes chronic inflammation and inflammation-associated colorectal cancer [36]. Therefore, mutant p53 not only confers drug resistance *via* upregulation of Nrf2 expression but it also may activate the NF-κB signaling pathway for additional enhancement of Nrf2 expression (Figure 1C).

The prevalence of “low” or “high” Nrf2 expression was not significantly revealed in p53-wild-type patients compared with p53-mutant patients. This conflicting may be due to wild-type p53 dysfunction by several mechanisms. For example, Nrf2 may promote MDM2 expression to increase p53 degradation [28]. An early report indicated that MDM2 mRNA expression may be used to predict p53 transcriptional function and patients' prognosis in NSCLC [37]. We thus evaluated MDM2 mRNA expression in p53-wild-type patients by real-time PCR in this study population and data showed that high Nrf2 mRNA expression was more commonly occurred in low-MDM2 mRNA tumors than in high-MDM2 tumors (Supplementary Table 3). Therefore, wild-type p53 dysfunction by different mechanisms may cause p53 wild-type patients with high Nrf2 mRNA tumors, and consequently resulting in the prevalence of “low” or “high” Nrf2 expression to be not significantly revealed in p53-wild-type patients.

ROS levels are tightly controlled by the Nrf2/Keap1 pathway [38]. However, an increased in Nrf2 transcription, that were triggered by oncogenes such as mutations in K-ras^G12D^ and BrafV619V and overexpression of Myc^ERT2^, promotes ROS detoxification and tumorigenesis [38]. K-ras increases Nrf2 gene transcription through a TPA response element located on the Nrf2 promoter [24]. In a mouse model of mutant K-ras^G12D^-induced lung cancer, suppressing the Nrf2 pathway with the chemical inhibitor Brusatol enhanced the antitumor efficacy of cisplatin [24]. These results strongly suggest that oncogenic K-ras promotes tumor malignancy as well as conferring cisplatin resistance in lung cancer through upregulation of Nrf2 transcription. However, K-ras mutations were not detected in this study population (*n* = 114, data not shown). In the present study, mutant p53 upregulated Nrf2 transcription by increased Sp1 binding to the Nrf2 promoter, thereby conferring cisplatin resistance. Consistent findings were also observed in wild-type p53 cells subjected to p53 silencing and in p53-null cells transfected with different mutant p53 expression vectors. We therefore suggest that mutant p53 may confer cisplatin resistance in lung cancer cells *via* upregulating Nrf2 transcription.

In summary, we provide evidence that upregulation of Nrf2 transcription by mutant p53 may confer cisplatin resistance, an unfavorable response to cisplatin-based chemotherapy, and poor outcomes in NSCLC patients. Therefore, we suggest that Bcl-2 antagonists might be helpful in improving cisplatin sensitivity and outcomes in p53-mutant NSCLC patients who harbor high-Nrf2 mRNA tumors.

## MATERIALS AND METHODS

### Study subjects

Lung tumor specimens were collected from 114 primary lung cancer at the Department of Thoracic Surgery, Taichung Veterans General Hospital (Taichung, Taiwan), between 1998 and 2004. Patients were asked to submit written informed consent and were approved by the Institutional Review Board (Institutional Review Board, Chung Shan Medical University Hospital, CSMUH No: CS11177). The tumor type and stage of each collected specimen were histologically determined according to the World Health Organization classification system. Cancer relapse data were obtained by chart review and confirmed by thoracic surgeons. Clinical parameters and OS and RFS data were collected from chart reviews and the Taiwan Cancer Registry, Ministry of Health and Welfare, Executive Yuan, ROC.

### Cell lines and culture conditions

H1299, H1355, H1650, H1975 and H358 cells were obtained from ATCC (Manassas, VA, USA) and were cultured as previously described (http://www.atcc.org). CL1-5 and CL3 cells were kindly provided by Dr. Pan-Chyr Yang (Department of Internal Medicine, National Taiwan University Hospital, Taiwan). TL4 cells were kindly provided by Dr. Ya-Wen Cheng (Graduate Institute of Cancer Biology and Drug Discovery, Taipei Medical University, Taipei, Taiwan). H1299 cells were grown in Dulbecco's modified Eagle's medium (Hyclone, Waltham, MA, USA) supplemented with 10% fetal bovine serum (Hyclone, Waltham, MA, USA). CL1-5, CL3, H1355, H1650, H1975, H358 and TL4 cells were grown in RPMI-1640 medium (Hyclone, Waltham, MA, USA) with 10% fetal bovine serum. The type of p53 mutations in eight cell lines has been listed in Supplementary Table 4. All cell lines were grown at 37°C in a 5% carbon dioxide atmosphere. These cells were cultured according to the suppliers' instructions and were stored used at passages 5 to 20. Once resuscitated, cell lines were routinely authenticated (once every 6 months; cells were last tested in December 2012) through cell morphology monitoring, growth curve analysis, species verification by isoenzymology and karyotyping, identity verification using short tandem repeat profiling analysis, and contamination checks.

### Plasmid constructs and transfection assays

Nrf2 cDNA was cloned into pcDNA3.1 Zeo(+) (Invitrogen, Carlsbad, CA, USA) by PCR amplification with newly created XhoI and BamHI sites attached on the Nrf2 5′ends of forward and reverse primers, using H1299 cDNA as template. The Nrf2 promoter reporter plasmid was constructed by inserting 1036, 740, and 229 bps KpnI/HidIII fragments into a KpnI/HidIII-treated pGL3 vector (Promega, Madison, WI, USA). The primer sequences are listed in Supplementary Table 5. The wild-type and mutant p53 constructs were kindly provided by Dr. Jiunn-Liang Ko (Institute of Medicine, Chung Shan Medical University, Taichung, Taiwan) [43]. The shRNA was purchased from National RNAi Core Facility, Academia Sinica, Taipei, Taiwan. Different concentrations of plasmids were transiently transfected into lung cancer cells (1 × 10^6^ cells) using the Turbofect reagent (Fermentas, Waltham, MA, USA). After 48 h, cells were harvested and whole-cell extracts were used for subsequent experiments.

### Real-time PCR analysis of mRNA expression levels

DNase I-treated total RNA (10 ng) was subjected to real-time PCR analysis with the Reverse Transcription Kit (Applied Biosystems, Foster City, CA), mRNA Assays (Applied Biosystems, Foster City, CA, USA), using a Real-Time Thermocycler 7500 (Applied Biosystems, Foster City, CA, USA). Glyceraldehyde 3-phosphate dehydrogenase (GAPDH) was used as the mRNA reference housekeeping gene. The primers used for real-time PCR analysis of mRNA expression are presented in Supplementary Table 5.

### Luciferase reporter assay

The luciferase reporter assay was conducted by transfecting appropriate numbers of cells with sufficient reporter plasmids, as determined from earlier studies [39].

### ChIP assay

ChIP analysis was performed as described previously [39]. The primer sequences are presented in Supplementary Table 5.

### 3-(4,5-cimethylthiazol-2-yl)-2,5-diphenyl tetrazolium bromide (MTT) cytotoxicity assay

The cell lines were cultured in 96-well flat-bottomed microtiter plates supplemented with RPMI 1640 and DMEM containing 10% heat-inactivated fetal bovine serum, 100 units/mL penicillin, and 100 units/mL streptomycin in a humidified atmosphere containing 95% air and 5% CO_2_ at 37°C in a humidified incubator. Before cisplatin treatment(0, 2, 4, 8, 16, 32 μM), the cells cultured in the exponential growth phase were pretreated with shRNAs, p53 and Nrf2 overexpression plasmid for 24 h. After 48 h incubation, the *in vitro* cytotoxic effects of these treatments were determined by MTT assay (at 570 nm).

### Statistical analysis

Statistical analysis was performed using the SPSS statistical software program Version 15.0 (SPSS Inc., USA). Survival plots were generated using the Kaplan-Meier method, and differences between patient groups were determined by a log-rank test. A multivariate Cox regression analysis was performed for overall survival and relapse free survival. The analysis was stratified for all known prognostic variables (age, sex, smoking status, tumor type, and stage) and for mRNA expression.

## SUPPLEMENTARY MATERIAL TABLES AND FIGURES


