# Supplementary material for: Mutant p53 confers chemoresistance in non-small cell lung cancer by upregulating Nrf2
Source: Oncotarget. 2015 Oct 19;6(39):41692–705. doi: 10.18632/oncotarget.6150 (PMC4747182; doi:10.18632/oncotarget.6150)
Supplement: Supplementary file 1 [file oncotarget-06-41692-s001.pdf]

Mutant p53 confers chemoresistance in non-small cell lung cancer by upregulating Nrf2

Supplementary Material

Supplementary Table 1. The mutation rates of Nrf2 and Keap1 in NSCLC patients.

	Case number	Mutation number	Mutation rate
Nrf2	114	3	2.6%
Keap1	114	2	1.8%

Supplementary Table 2. Chemotherapeutic agents used in this study population.

**Chemotherapeutic agents**	Patient number (%)
Cisplatin	5 (8.3)
Cisplatin+ gemzar	44 (73.3)
Cisplatin+ vp16	5 (8.3)
Cisplatin+ taxol	5 (8.3)
Cisplatin+ mitomycin C	1 (1.7)

Patients were collected from 1996 to 2003

Supplementary Table 3. Relationships between MDM2 and Nrf2 mRNA expression in p53 wild-type NSCLC patients.

Characteristic	Case no.	MDM2		*P*
		Low (%)	High (%)	
	85	43 (50.6)	42 (49.4)	
Nrf2				
Low	48	35 (72.9)	13 (27.1)	0.001
High	37	8 (21.6)	29 (78.4)	

Supplementary Table 4. List of p53 status in p53 mutation cell lines.

Cell line	p53 status
H1355	P72R E285K
H358	null
CL1-5	R248W
H1299	null

Supplementary Table 5. List of primer sequences used in the present study.

Target gene	Sequence
**Real-time PCR**	
GAPDH Forward	5'-GGAGCCAAAAGGGTCATCATC-3'
GAPDH Reverse	5'-GATGGCATGGACTGTGGTCAT-3'
Nrf2 Forward	5'-GTGAAGGCGCTATTTGGCG-3'
Nrf2 Reverse	5'-GGTCCATAGTGACGGTCAGGT-3'
Bcl-2 Forward	5'-CTGTGGATGACTGAGTACC-3'
Bcl-2 Reverse	5'-CAGCCAGGAGAAATCAAAC-3'
Bcl-xL Forward	5'- GCTGGGACACTTTTGTGGAT-3'
Bcl-xL Reverse	5'- TGTCTGGTCACTTCCGACTG-3'
Nrf2 promoter reporter plasmid	
-1036 Forward	5'-GGTACCTCGTTGATTCCACAGCATTT-3'
-740e Reverse	5'-GGTACCCTGCCGGAGCTGTCCACATCTC-3'
-229 Forward	5'-GGTACCGAAGGAAGGGCCCGGACTCTTG-3'
Reverse	5'-AAGCTTGAGCTGTGGACCGTGTGTTGGG-3'
Mutated p53 binding site Forward	5'- CCCTGATTTGGAGTTGCAGAACTTTTCTCTGCTTTTATCTCACTTTACCG-3'
Mutated p53 binding site Reverse	5'- CGGTAAAGTGAGATAAAAGCAGAGAAAAGTTCTGCAACTCCAAATCAGGG-3'
Mutated Sp1 binding site Forward	5'- GGCGCCAGCCGGGGTTGTGTGGGCTAAAGATTTGGA-3'
Mutated Sp1 binding site Reverse	5'-TCCAAATCTTTAGCCCACACAACCCCGGCTGGCGCC-3'
ChIP primer	
Forward	5′-GAATGGAGACACGTGGGAGT-3′
Reverse	5′-CCTTGCCCTGCTTTTATCTCA-3′
Nrf2 expression vector plasmid	
Forward	5'-CTCGAGATGATGGACTTGGAGCTGCCGCC-3'
Reverse	5'-GGATCCCTAGTTTTTCTTAACATCTGGCT-3'
**RNAi target**	
Shp53	5'- CACCATCCACTACAACTACAT-3'
shNrf2	5'- CCGGCATTTCACTAAACACAA-3'

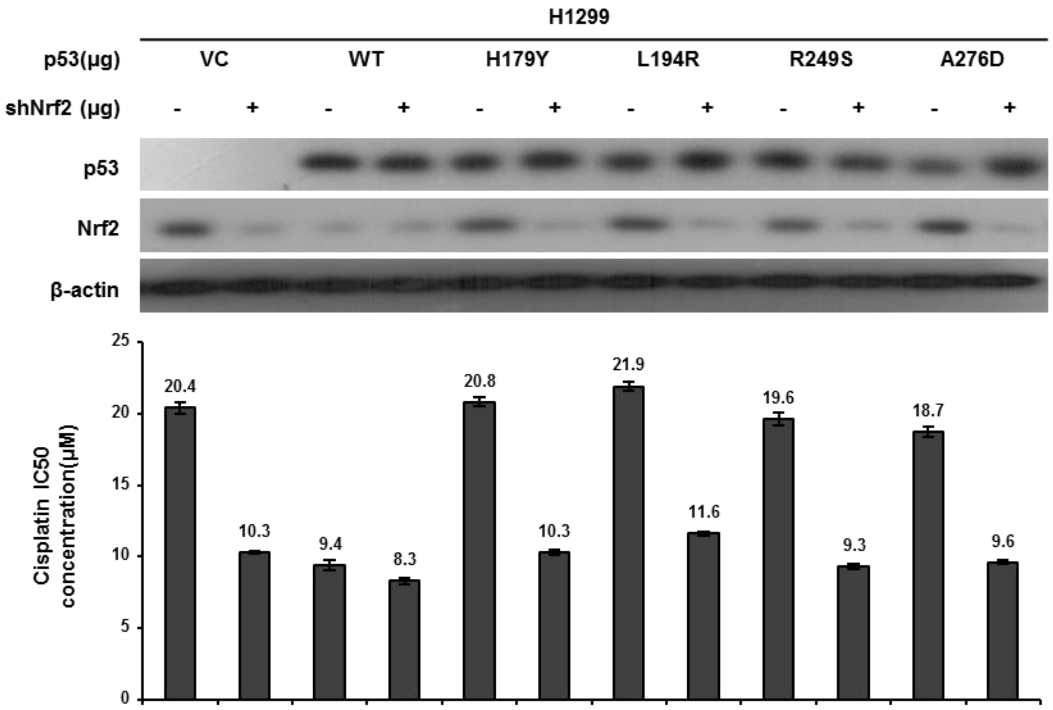

Supplementary Figure 1. The H1299 cells were transfected with wild-type p53, mutant p53 and/or shNrf2 plasmid. After 24 h, the cells were treated with or without cisplatin for an additional 48 h for MTT assay. The cell lysates were separated by SDS-PAGE for the evaluation of p53, Nrf2, expression by specific antibodies using western blotting. The MTT assay was used to determine the IC50 of cisplatin.

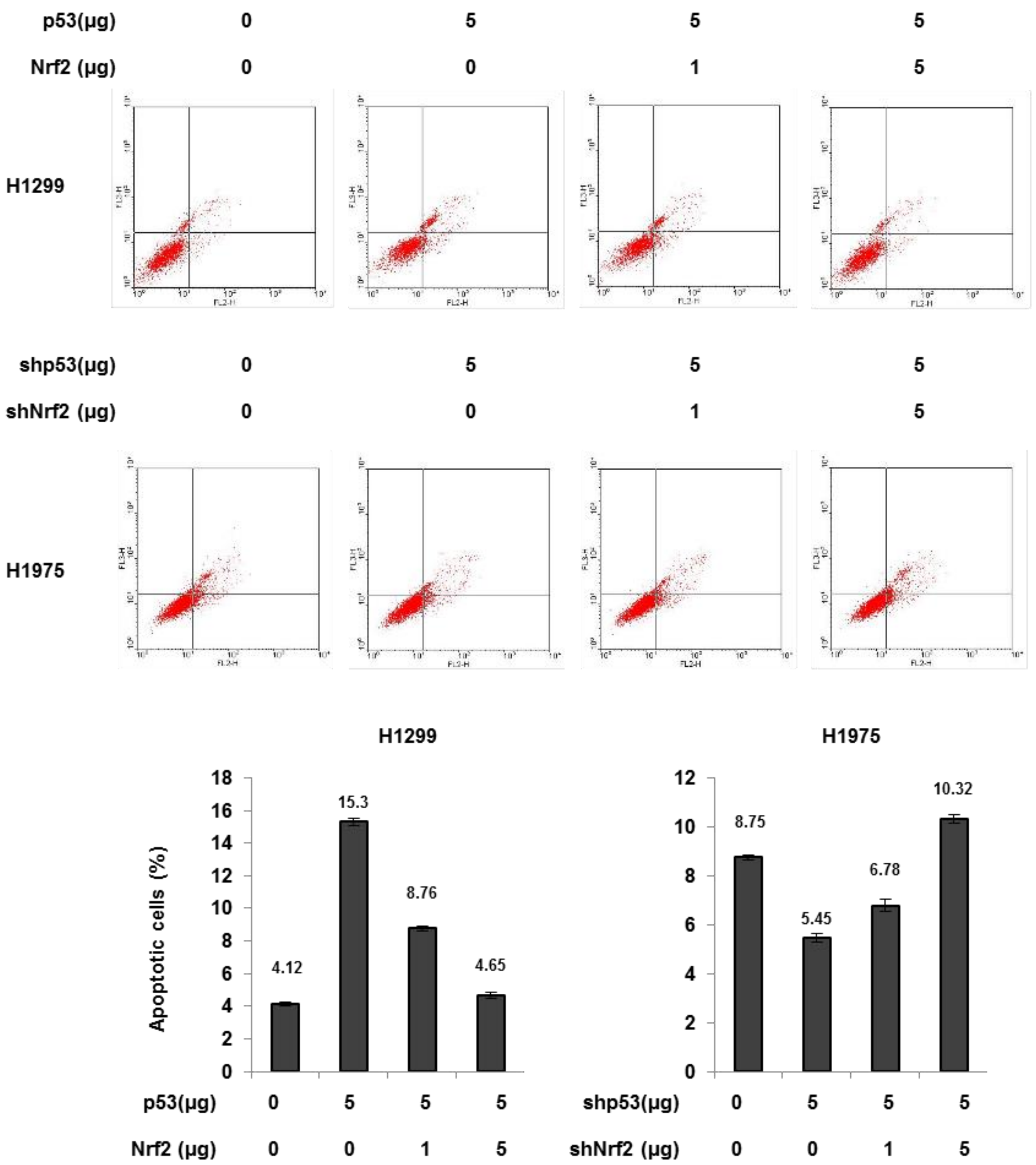

Supplementary Figure 2. Flow cytometric analysis of apoptosis with annexin V/PI staining. H1299 cells were transfected with p53 and/or Nrf2 plasmid. H1975 cells were transfected with shp53 and/or shNrf2 plasmid. The cells were then subjected to annexin V and PI staining, followed by a flow cytometry. Percentage of apoptotic cells including with the Annexin V+/PI− population (early apoptosis) plus Annexin V+/PI+ (late apoptosis/secondary necrosis) was summarized by a flow cytometric analysis. Data are expressed as means±s.d., n=3.

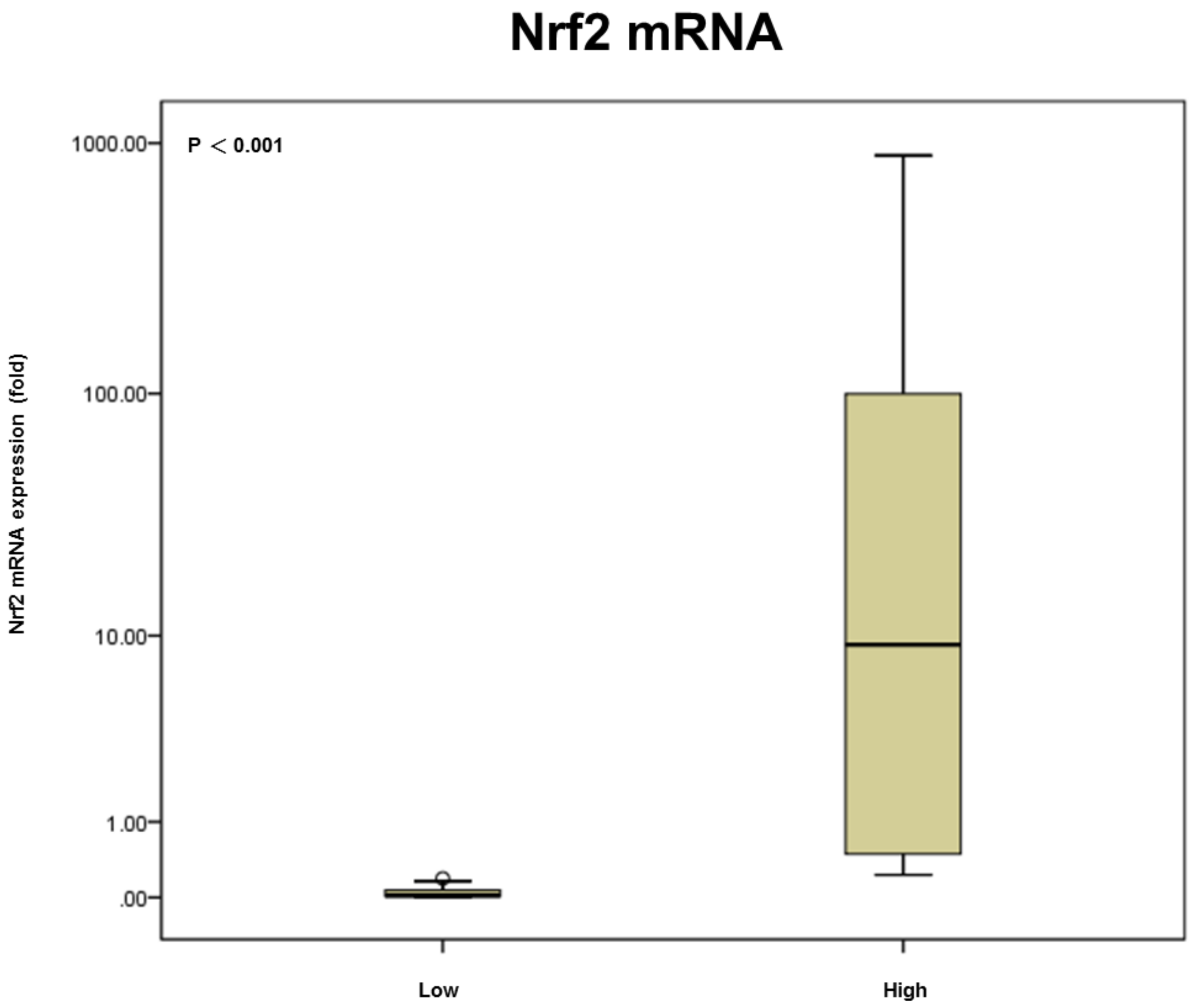

Supplementary Figure 3. The Box plot analysis for Nrf2 mRNA expression levels between low and high subgroups in lung cancer patients. The charts show the expression of relative quantification values.
